# Editorial: Zoonotic diseases originating from wildlife: Emergence/re-emergence, evolution, prevalence, pathogenesis, prevention, and treatment

**DOI:** 10.3389/fmicb.2023.1165365

**Published:** 2023-03-14

**Authors:** Hongliang Chai, Quan Liu, Natasha N. Gaudreault, Wanjiang Zhang

**Affiliations:** ^1^College of Wildlife and Protected Area, Northeast Forestry University, Harbin, China; ^2^Department of Infectious Diseases and Center of Infectious Diseases and Pathogen Biology, Key Laboratory of Organ Regeneration and Transplantation of the Ministry of Education, Key Laboratory of Zoonotic Diseases, The First Hospital of Jilin University, Changchun, China; ^3^Department of Diagnostic Medicine/Pathobiology, College of Veterinary Medicine, Kansas State University, Manhattan, KS, United States; ^4^State Key Laboratory of Veterinary Biotechnology, Harbin Veterinary Research Institute, Chinese Academy of Agricultural Sciences, Harbin, China

**Keywords:** zoonotic diseases, wildlife, evolution, prevalence, pathogenesis, prevention, treatment, emergence/re-emergence

Wild animals carry a variety of pathogens and can serve as natural reservoirs of pathogens because they live in complex environments. Wildlife diseases not only pose a serious threat to animal health, especially rare and endangered wild animals, but also cause important zoonoses that threaten public health, such as AIDS, Ebola, avian influenza, and others. It has been reported that 60.3% of emerging human infectious diseases are animal-borne, of which 71.8% have originated from wildlife. In addition, due to the habits of wildlife migration, wild animals and birds are major disease spreaders, as they can transmit many zoonotic diseases across regions and country borders, intercontinentally and globally. Meanwhile, many mosquitoes and ticks carried by wildlife are also major vectors of diseases (vector-borne diseases), facilitating disease spread, causing huge economic losses to poultry and livestock, and bringing major public health problems to human beings. With this context, we launched this Research Topic on 10 January 2022. Frontiers in Microbiology published 12 articles, involving 139 authors from nine countries. Despite the diversity of 10 zoonotic diseases, the contributions fall into three research areas: viral diseases, bacterial diseases, and parasitic diseases.

## Viral diseases

The first area of research, viral diseases, included avian influenza, AIDS, COVID-19, parvovirus infection, hemorrhagic fever with renal syndrome (HFRS), Monkeypox (MPX), and tick-borne encephalitis virus. Emerging infectious diseases are one of the main threats to public health, with the potential to cause a pandemic when the infectious agent manages to spread globally. Miranda et al. reviewed three major pandemics: the influenza pandemic, the human immunodeficiency virus (HIV) pandemic, and the COVID-19 pandemic. They analyzed these pandemics' historical and epidemiological contexts and the determinants of their emergence. Furthermore, they compared pharmaceutical and non-pharmaceutical interventions that had been used to slow down these three pandemics and focus on the technological advances that were made in the progress. Finally, they discussed the evolution of epidemiological modeling, which has become an essential tool to support public health policymaking and they discuss it in the context of these three pandemics. While these pandemics were caused by distinct viruses that started in different periods and different regions of the globe, their work showed that many of the determinants of their emergence and countermeasures used to halt transmission were common. Therefore, it is important to further improve and optimize such approaches and adapt them to future-threatening emerging infectious diseases.

Since it was first identified in 1956, the H11 subvariant influenza virus has been reported worldwide for several decades. Jiang, Li, et al. isolated a strain of H11N3 avian influenza virus (AIV) from poultry feces in live poultry markets in the southeast coastal region of China. Considering that H11 subvariants were known to cause human infections and to enrich the knowledge of the H11 subvariant avian influenza virus, genetics, pathogenicity, and transmissibility of the isolate were studied. Phylogenetic analysis indicated that the H11N3 isolate was of Eurasian origin and carried genes that were closely related to duck H7N2 and H4N6. The receptor-binding preference analysis revealed that the H11N3 isolate only acquired a binding affinity for avian-derived receptors. In the respiratory system of mice, the isolated virus could directly infect mice without adaptation. In addition, results from transmission experiments and the detection of antibodies in guinea pigs demonstrated that the H11N3 influenza virus can efficiently transmit through the respiratory tract in mammalian models. Direct infection of the H11N3 influenza virus without adaptation in mouse models and aerosol transmission between guinea pig models confirmed its pandemic potential in mammals, underscoring the importance of monitoring rare influenza virus subtypes in future studies.

To date, there have been three epidemic waves of H5N8 avian influenza worldwide. The current third epidemic wave began in October 2020 and has expanded to at least 46 countries. Jiang, Liu, et al. conducted active and passive surveillance to monitor H5N8 viruses from wild birds in China. Genetic analysis of 10 H5N8 viruses isolated from wild birds identified two different genotypes. Animal challenge experiments indicated that the H5N8 isolates were highly pathogenic in chickens, and mildly pathogenic in ducks, while pathogenicity varied in Balb/c mice. Moreover, there were significant differences in antigenicity as compared to the Re-11 vaccine strain, and vaccinated chickens were not completely protected against the high dose of the H5N8 virus. With the use of the new matched vaccine and increased poultry immune density, surveillance should be intensified to monitor the emergence of mutant strains and potential worldwide spread *via* wild birds.

Avian-to-mammal transmission and mammalian adaptation of AIV are threats to public health and are of great concern. The H3 subtype of the influenza virus has low pathogenicity and is widely distributed in humans, canines, equines, and avians. In 2018–2019, Yao et al. isolated six H3N2 subtype influenza viruses from 329 samples acquired from ducks in China as part of an ongoing virus surveillance program. One strain of the H3N2 virus was a novel reassortant influenza virus containing HA and PB2 segments from the canine H3N2 virus. The findings suggested that the viruses studied have undergone multiple reassortment events. Their results provided a framework for understanding the molecular basis of host-range shifts of influenza viruses and indicated that dogs were potential “mixing vessels” for influenza virus transmission.

COVID-19 has emerged as a major public health challenge worldwide. A comprehensive understanding of clinical characteristics and immune responses in COVID-19 asymptomatic carriers and symptomatic patients was of great significance to the countermeasures of COVID-19 patients. Li et al. described the clinical information and laboratory findings of 43 individuals from Hunan Province, China, including 13 COVID-19 asymptomatic carriers, 10 symptomatic patients, and 20 healthy controls from 25 January to 18 May 2020. Their results showed that for cytokines, significantly higher Th1 cytokines including IL-2, IL-8, IL-12p70, IFN-γ, and TNF-α as well as Th2 cytokines including IL-10 and IL-13 were observed in symptomatic patients compared with asymptomatic carriers. Compared with symptomatic patients, higher N-specific IgG4/IgG1 ratios and RBD-specific/N-specific IgG1 ratios were observed in asymptomatic carriers. Comparable nAbs were detected in both asymptomatic carriers and symptomatic COVID-19 patients. In the symptomatic group, nAbs in patients with underlying diseases were weaker than those of patients without underlying diseases. Their retrospective study enriches and verifies the clinical characteristics and serology diversities in COVID-19 asymptomatic carriers and symptomatic patients.

The fact that wild felines are carriers of pernicious infectious viruses should be a major concern due to the potential cross-species transmission between the felines and human or domestic animals. Huang et al. screened four infectious viruses: feline parvovirus (FPV), feline coronavirus (FCoV), canine distemper virus (CDV), and influenza A virus (IAV) in the blood samples of 285 captive Siberian tigers and the spleen samples from two deceased lions, which were collected from 2019 to 2021 in three Siberian Tigers Parks from the northeast of China. The results showed that FPV circulated in the captive Siberian tigers and lions in northeastern China, and this provided valuable information for the study of FPV epidemiology in wild felines.

To analyze the clinical significance of serum ferritin, procalcitonin (PCT), and C-reactive protein (CRP) in hemorrhagic fever with renal syndrome patients, Che et al. analyzed the demographical, clinical, and laboratory data in 373 HFRS patients in northeastern China retrospectively. The levels of serum ferritin and PCT in severe patients (*n* = 108) were significantly higher than those in mild patients (*n* = 265, *p* < 0.001) and associated with HFRS severity. The serum ferritin level in non-survivors (*n* = 14) was significantly higher than in survivors (*n* = 359, *p* < 0.001). They found that serum ferritin and PCT had a robust association with HFRS severity and mortality, which might be promising predictors, and CRP was an effective biomarker to assess bacterial co-infection in HFRS.

Monkeypox was first reported in humans in 1970, and outbreaks were restricted and highly localized to endemic regions of western and central Africa. However, after the first reported case in the UK in early May 2022, Karim et al. believed that the pattern of epidemic spreading in the geographical regions was much larger compared to the past, posing a risk that MPX might become entrenched beyond endemic areas. This virus is less transmissible than SARS-CoV-2, as it is transmitted mainly through personal, close, often skin-to-skin contact with infectious MPX rash, body fluids, or scabs from an individual with MPX. Infections usually present with chills, fever, fatigue, muscle aches, headache, sore throat, skin lesions, and lymphadenopathy. Currently, there are no antivirals approved for MPX. However, an antiviral drug called “tecovirimat” which was approved for the treatment of smallpox has been made accessible to treat MPX. Moreover, to prevent MPX, there are two vaccines available that are approved by FDA: Bavarian Nordic JYNNEOS and ACAM2000 vaccine. Contact tracing is absent in the case of an MPX outbreak and there is a lack of information from the data systems rapid manner. Additionally, test capacity needs to be increased. Like SARS-CoV-2, global demand for vaccines for the MPX outbreak far exceeds availability.

The geographic range of tick-borne encephalitis virus (TBEV) and the human incidence is increasing throughout Europe putting many non-endemic regions and countries at risk of outbreaks. In the spring of 2020, there was an outbreak of TBE in Ain, Eastern France, where the virus had never been detected before. Gonzalez et al. investigated the suspected farm using an integrative One Health approach. They confirmed the alimentary origin of the TBE outbreak and witnessed in real-time the seroconversion of recently exposed individuals and the excretion of the virus in goat milk. In addition, they identified a wooded focus area where and around which there was a risk of TBEV exposure, provided the first TBEV isolate which was a source of dietary contamination in France, obtained its full-length genome sequence, and found that it did not cluster very closely neither with the isolate circulating in Alsace nor with any other isolate within the European lineage. TBEV is now a notifiable human disease in France, which facilitates surveillance of TBEV incidence and distribution throughout France.

## Bacterial diseases

The bacterial diseases in our Research Topic included *Bartonella* infection and *pseudotuberculosis*. Rodents are the primary natural reservoirs of *Bartonella* spp. and some are zoonotic causative agents. Hence, surveillance of *Bartonella* sp. infection in rodents is very important for the prevention of human bartonellosis. Jian et al. captured rodents and collected their spleen samples for *Bartonella* sp. DNA detection and identification by amplifying the 16S rRNA, gltA, and ftsz genes using semi-nested polymerase chain reaction (PCR). The results indicated that *Bartonella* sp. DNA was detected in seven *Rattus norvegicus* individuals with a detection rate of 6.7% in Chengde City, while bacterial DNA in 31 *Apodemus agrarius* individuals with a detection rate of 28.4% in Handan City. The DNA detection rate across the rodents' genders and ages was not statistically significant. Furthermore, sequence analysis of the above-mentioned three genes demonstrated that at least eight *Bartonella* species were circulating in Hebei Province, of which three, including *Bartonella rattimassiliensis, Bartonella grahamii*, and *Bartonella tribocorum* are human pathogens, thus suggesting the existence of a major public health risk. Overall, these results revealed the detection rate and genetic diversity of *Bartonella* species infection in rodents in Hebei Province, which could be potentially helpful for the prevention of bartonellosis caused by rodent-associated *Bartonella* species. Their study highlighted the urgent need for the surveillance of *Bartonella* infection in rodents and ectoparasites that affect both rodents and humans and could cause fever of unknown origin or endocarditis.

*Yersinia pseudotuberculosis* is a foodborne zoonotic bacterium that is pathogenic to guinea pigs, rabbits, and mice. It also causes *pseudotuberculosis* in humans. Dong et al. found out that Ebselen (EbSe) exhibited synergistic antibacterial activity with silver nitrate (Ag+) against *Y. pseudotuberculosis* YpIII strain with high efficacy *in vitro* using UV-vis, DTNB, LSCM, FCM, ETM, and WB assays. The depletion of total glutathione (GSH) amount and inhibition of thioredoxin reductase (TrxR) activity in the thiol-dependent redox system revealed the destructiveness of EbSe-Ag+-caused intracellular oxidative stress. Furthermore, a YpIII-caused mice gastroenteritis model was constructed, and EbSe-Ag+ significantly reduced bacterial loads with low toxicity, downregulated the expression levels of interferon (IL)-1β and tumor necrosis factor-α, and upregulated the expression level of IL-10 on-site, demonstrating the *in vivo* antibacterial activity and immune-modulatory property of EbSe-Ag+. Collectively, these results provided academic fundamentals for further analysis and development of EbSe-Ag+ as the antibacterial agent for *pseudotuberculosis* control.

## Parasitic diseases

The third area of research was parasitic diseases. *Echinococcus multilocularis*, the causative agent of alveolar echinococcosis (AE), severely threatens human health and livestock farming. The first line of chemotherapeutic drug for AE is albendazole, which limits the rapid extension of *E. multilocularis* metacestodes, but is rarely curative for AE, with severe side effects in long-term use. Thus, the development of new anti-echinococcal drugs is mandated. Pseudolaric acid B (PAB) has long been used to treat fungal-infected dermatosis and exerted anti-tumor, -fertility, -angiogenesis, -tubulin, and antiparasitic activity. However, the effect of PAB against *Echinococcus* spp. remains unclear. Gao et al. found that after exposure to PAB at 20 μg/ml, a significant reduction of the survival rate and substantial ultrastructural destructions in *E. multilocularis* protoscoleces were observed *in vitro*. Furthermore, the wet weight of *E. multilocularis* cysts in the infected mice was significantly decreased after treatment with PAB (40, 20, or 10 mg/kg) for 12 weeks. Meanwhile, a significant increase of both protein and mRNA expression of transforming growth factor-beta 1 (TGF-β1) was detected in the serum and liver of the infected mice, whereas PAB administration lowered its expression significantly. The toxicity tests demonstrated that PAB displayed lower cytotoxicity to human liver and kidney cells (HL-7702 and HK-2 cell) with IC_50_ = 25.29 and 42.94 μg/ml than albendazole with IC_50_ = 3.71 and 21.22 μg/ml *in vitro*, and caused lower hepatoxicity and nephrotoxicity in mice than ABZ. Their findings indicated that PAB possesses a potent anti-echinococcal effect with lower toxicity than albendazole, implying it is a potential chemotherapeutic agent for AE. Additionally, their study demonstrated that the suppressive effect of PAB on the parasite may involve the down-regulation of TGF-β1 signaling.

In conclusion, these articles find that the spillover of pathogens from wildlife to domestic animals or humans can lead to the rapid evolution of the pathogen. Under the immune stress in new hosts, pathogens can evolve and acquire new biological properties which may pose a serious threat to the health of domestic animals and humans. Pathogens carried by wild animals and birds are not only transmitted across proximal hosts but can also spread over wide ranges geographically *via* wildlife migration. Understanding the risks of wildlife-borne zoonotic diseases is necessary to increase awareness and facilitate the application of preventive and control measures to reduce disease spread.

## Author contributions

All authors listed have made a substantial, direct, and intellectual contribution to the work and approved it for publication.

